# C-reactive protein diagnostic test uptake in primary care: a qualitative study of the UK’s 2019–2024 AMR National Action Plan and lessons learnt from Sweden, the Netherlands and British Columbia

**DOI:** 10.1136/bmjopen-2024-095059

**Published:** 2025-08-31

**Authors:** Rebecca E Glover, Agata Pacho, Nicholas Mays

**Affiliations:** 1 Health Services Research and Policy, London School of Hygiene & Tropical Medicine, London, UK

**Keywords:** Health policy, INFECTIOUS DISEASES, Primary Health Care

## Abstract

**Abstract:**

**Objectives:**

This study compares the implementation of C-reactive Protein (CRP) testing to distinguish between acute bacterial and viral infections in primary care in three WHO European region countries (Sweden, the Netherlands and the UK) and one WHO Americas region country (Canada) to generate insights that health services can use to guide decision-making on CRP test implementation.

**Setting:**

Three settings were included: (1) national-level policy interviewees in England, (2) front-line antibiotic prescribers operating in primary care in England and (3) national-level policy interviewees in Canada, Sweden and the Netherlands.

**Participants:**

We conducted in-depth qualitative interviews with 35 UK antimicrobial resistance policy-makers, clinicians and allied medical professionals. We also interviewed 13 national-level diagnostic test experts in Canada, Sweden, the Netherlands and the UK. We transcribed, thematically coded and analysed the data, using the NASSS framework for analysing technology-supported healthcare change.

**Results:**

There were multiple barriers and facilitators to CRP test implementation. The Netherlands was seen as the country with the most successful implementation of CRP, with enabling IT, financing and quality assurance infrastructure alongside strong guideline adherence to prevent overtesting. By contrast, Swedish interviewees reported ‘CRP-itis’, or widespread, guideline-discordant testing. UK uptake, contingent on local-level commissioning, has stalled amid mixed professional views. Canadian professional views are sceptical, and uptake plans are nearly non-existent. The NASSS themes emerging as the strongest facilitator or barrier to diagnostic uptake were the organisation and wider systems, with some reflections emerging on the limitations of NASSS to capture interface issues emerging from the analysis.

**Conclusions:**

Health services considering CRP test adoption in primary care need to be mindful that, where concerns about the value proposition of the technology exist among policy-makers and professionals, CRPs are unlikely to be implemented; where there are discordant practices between policymakers/guidelines and clinicians, adoption of CRP testing may increase both antibiotic prescribing and costs to the health system. Where there is local-level commissioning of diagnostic tests, uptake may stagnate; and where implementation succeeds, it may be to the detriment of the public purse.

**STRENGTHS AND LIMITATIONS OF THIS STUDY**

- This is a qualitative study arising from an implementation evaluation of the UK Antimicrobial Resistance National Action Plan; this means that we had access to high-level policymakers across the UK.
- However, because the evaluation was primarily focused on the UK policy context, international interviewees were fewer, and constrained to areas where we and policymakers considered that lessons could be learnt from other policy contexts.
- Data saturation was achieved in the UK interviews, but there were relatively fewer interviewees internationally; these were supplemented by document analysis and targeted to particular areas of policy. This limitation was mitigated by the fact that the primary unit of analysis is the UK context, and the recommendations for actions are limited to the UK context.

## Introduction

### Antimicrobial resistance

 Antimicrobial resistance (AMR) is a global policy priority. Both the United Nations General Assembly (UNGA) and World Bank in 2024 formally recommended the adoption of more rapid diagnostic tests, ideally at point of care, to reduce antibiotic prescribing, which is likely to contribute to a reduction in AMR.^1 2^ The emergence of resistance in bacteria is a natural phenomenon that has accelerated in response to the use of antibiotics in medicine and agriculture and their release into the environment. The rise in multidrug-resistant infections leads to significant morbidity, mortality and economic loss, with the number of deaths directly attributable to AMR exceeding 1.7 million annually; one-fifth of which are in under-5s.^3 4^ The growth of AMR has led countries to implement extensive AMR policies across human, animal and environmental health, covering areas as diverse as infection prevention and control, wastewater surveillance, the banning of antibiotics as growth promoters, financial incentives for prescribers and many more.^5 6^

### C-reactive protein testing as an AMR policy

C-reactive protein (CRP) tests are one often-touted test which is increasingly affordable and can often distinguish between bacterial and viral infections.^7 8^ Various high-income countries have issued guidance about CRP test usage in primary care, often in cases of acute lower respiratory tract infection (LRTI) with uncertainty surrounding a diagnosis of pneumonia. The National Institute for Health and Care Excellence (NICE) guidance in the UK recommends CRP testing for pneumonia in adults: ‘for people presenting with symptoms of LRTI in primary care, consider a point-of-care CRP test if after clinical assessment a diagnosis of pneumonia has not been made and it is not clear whether antibiotics should be prescribed.’^9^ The Dutch College of General Practitioners recommends CRP testing for ‘acute cough’ in adults, only in cases of genuine clinical uncertainty for pneumonia, having revised their guidance in 2024, saying ‘a CRP test is usually not necessary in patients with acute coughing, as the GP can often accurately assess whether the patient has pneumonia based on the clinical picture. A CRP test is only useful in patients without risk factors for severe outcomes where there is doubt about pneumonia. We do not recommend CRP testing in children with acute cough.’^10^ Swedish guidance on community use of CRP testing similarly hinges on whether diagnosis is unclear, with potential for pneumonia, and also on the duration of symptoms.^11^ The Swedish government expects ‘quality-assured and adequate diagnostics with the shortest response time possible to underlie the prescribing of antibiotics, regardless of the form of care, in order to avoid unnecessary and incorrect treatment and to enable follow-up’.^12^ Furthermore, the Swedish government has invested in diagnostic testing for microbiological surveillance.^13^ Swedish government documents describe how ‘[d]iagnostics with routine taking of samples forms the basis of surveillance in the Swedish healthcare system. This, combined with automated, daily collation of laboratory results, produces clinically relevant resistance data’(15).

However, while the Netherlands has embedded CRP tests into primary care, the UK and Sweden face serious implementation challenges.^14^ Still other jurisdictions, such as the province of British Columbia (BC) in Canada, have decided against any routine implementation of such tests for acute LRTIs.

### UK AMR policy and CRP guidance

In 2019, the Department of Health and Social Care released the UK AMR National Action Plan (NAP),^15^ which outlines ambitions and actions over a 5-year period and supports the UK’s 20-year vision for AMR.^16^ The UK AMR NAP proposes that rapid and accurate tests at the point of care can help reduce AMR pressures while also improving patient safety and health outcomes by ensuring the right patient management. One of the commitments expressed was to ‘be able to report on the percentage of prescriptions supported by a diagnostic test or decision support tool by 2024’.^15^ ^(p.5)^ Encouraging the use of diagnostic tests designed to distinguish viral from bacterial infections, such as CRP testing, was thought to enable prescribers more accurately to decide on an antibiotic prescription (or none), and thus decrease inappropriate prescribing. There was also an ambition to distinguish low-severity from high-severity infections by using CRP to allow safe withholding of antibiotics from patients with self-limiting mild bacterial infections. Despite this commitment, uptake of CRP testing in UK primary care remains sparse.

As a part of the wider evaluation of the UK’s AMR NAP, this two-part qualitative study first aimed to understand why uptake of CRP tests in the UK had been so limited, and then to identify any lessons that could be derived from studying experts’ views of CRP testing in comparable health systems in three WHO European region countries (Sweden, the Netherlands and the UK) and one WHO Americas region country (Canada). We aimed to identify the barriers and facilitators to CRP testing in each country guided by the NASSS framework, and thus better guide CRP implementation in primary care in the UK and in health systems around the world.

## Methods

### Selection of comparator countries

This qualitative study was a part of a wider evaluation of the implementation of the UK’s AMR NAP.^17^ Comparator countries were selected on the basis of having sufficiently similar demographics, health systems and antibiotic usage rates to the UK and were: Sweden, the Netherlands and Canada.

### Qualitative interviews: sampling

Interviewees were selected from five regions in the UK: the North-East of England, North-West London, Aneurin Bevan Local Health Board in Wales, NHS Lothian in Scotland, and two areas piloting community pharmacy dispensing of antibiotics for minor ailments that were combined (Cornwall and the Isle of Wight). These sites were selected purposively to include a mix of urban and rural environments, areas of high tourism, and areas with and without difficulty recruiting and retaining professional clinical staff. Interviewees were purposively sampled based on expertise in AMR policy-making, clinical expertise or diagnostic expertise. Among the local healthcare professionals we interviewed, those who had technical information and opinions on the introduction and adoption of rapid diagnostic tests (including CRP tests) comprised consultant microbiologists, GPs, nurses, consultant pharmacists and pharmacists working in the ICB. For the international comparison that followed, we purposively selected senior policy experts within the AMR teams in regional (Canada) or national governments (UK, Sweden), or clinical thought leaders also working in academia, who have diagnostic policy experience in AMR (Canada, Netherlands).

### Development of the interview guide

We created a semistructured interview guide based on the study’s objectives and the Non-adoption, Spread, Scale-up and Sustainability (NASSS) framework (see table 1).^18 19^ The NASSS framework departs from classical Diffusion of Innovation theory by first considering the complexity within implementation processes, as well as the embedding and adaptation process over time.^20^ Next, rather than innovation ‘success’ being defined narrowly as adoption of a technology, the NASSS framework considers that success can also mean non-adoption of a novel technology due to reasonable concerns related to one or more of the factors listed in table 1.^19^ We paid particular attention to the final six domains within the NASSS framework, since across all country contexts, we focused on CRP guidance to inform diagnosis of pneumonia in primary care. The limitations of the NASSS framework are that it can be challenging to identify all the key domains among the ‘wider system’ to interrogate, such as commercial interests and power relationships; other more critical, sociological models might be better suited and more sensitive to these questions. Moreover, as with any framework, the interface themes that sit at/among categories can be problematic to operationalise. However, the NASSS framework works well to characterise the UK context, where successive AMR policies have recommended CRP test uptake in certain primary care contexts, but with little movement in this regard.

**Table 1:** The NASSS framework adapted from Greenhalgh *et al*^19^

NASSS domain	Explanation of domain	Covered in this study?
1	The illness or condition.	N
2	The technology, its material properties, knowledge needed to use it, knowledge it brings into play and intellectual properties.	Y
3	The value proposition—both supply-side (value to the developer) and demand-side (value to the patient, healthcare system and taxpayer or insurer). Can include business plans (eg,when efficacy or cost-effectiveness studies are unavailable or contested).	Y
4	The adopter system: the staff, patients and carers who will be expected to use the technology. Can be issues when roles and practices assumed by the technology threaten professional jurisdictions.	Y
5	The organisation(s), for example, the organisation’s ability to innovate, readiness for technology, balance of supporters and opponents, the nature of adoption and funding decisions, which can be more complex if it depends on interorganisational work and cross-system savings.	Y
6	The wider system, including the policy context, support from regulatory or professional bodies, public perceptions, networking and so on.	Y
7	Embedding and adapting over time, including inability to adapt to changing contexts, or the organisation’s inability to withstand (exogenous) shocks and setbacks through learning and adaptation.	Y

NASSS, Non-adoption, Spread, Scale-up and Sustainability.

### Interview data collection

Setting: First, we conducted in-depth qualitative interviews with 35 UK AMR policy-makers, clinicians and allied medical professionals from January to June 2023. All interviews were conducted online. Next, in order to probe the ‘solutions’ and examples of ‘success’ described by local prescribers in part one, we conducted 13 interviews with international AMR and diagnostics experts who had previously or currently worked on the UK, Swedish, Dutch, and Canadian context, from July to October 2023 (see table 2). Since the Canadian system is federal, with the provinces having considerable health system jurisdiction and autonomy, we focused at the provincial level on BC. The interviews were conducted in English, and all interviews lasted between 20 and 90 min, with most between 30 and 60 min.

**Table 2:** Overview of interviewees by WHO region and country

Study part	WHO region	Country	Scale of decision-making	Number of interviewees
1	European Region (EURO)	UK	Local	35
2	European Region (EURO)	UK	National	4
		Sweden	National	4
		The Netherlands	National	1
			Academic	1
	Region of the Americas (PAHO)	Canada	Provincial academic	2
				1

All interviews with international AMR and diagnostics experts were conducted by REG online, while the UK interviews were conducted by REG, NM, AP and several other researchers mentioned in the acknowledgements. Multiple researchers coded the UK interviews as relevant to diagnostics, and then those sections of the interviews were reanalysed using the NASSS framework by REG and AP. REG and AP are Canadian and Polish white female researchers in their 30s and 40s, working in UK health services research and living in the UK.

Interviewees were emailed, and given a participant information sheet. If they chose to participate, then they provided oral and written consent. Interviews were then undertaken on Microsoft Teams or Zoom, recorded and transcribed verbatim. Interviews were all held online, though in-person interviews had been planned in 2019 pre-COVID-19.

Pseudonymisation was a risk within this interviewee pool, since participants’ job titles or initials by each UK case study site would be sufficient to risk pseudonymisation. We have therefore left the interviewee number and country, which is considered best practice where pseudonymisation was a risk in similar qualitative research settings.^21^ Because there are two types of interviewees from the UK, where interviewees were local-level prescribers, they are attributed as (interviewee #); where they are national-level policy-makers, they are attributed as (UK#). Interviewees discussing Swedish, Canadian and Dutch national-level AMR policy are attributed as Sw#, Ca# and NL#, respectively. Because of their expertise, they also held multiple affiliations, including international affiliations, so cannot be further identified.

### Data analysis

**Figure 1:**
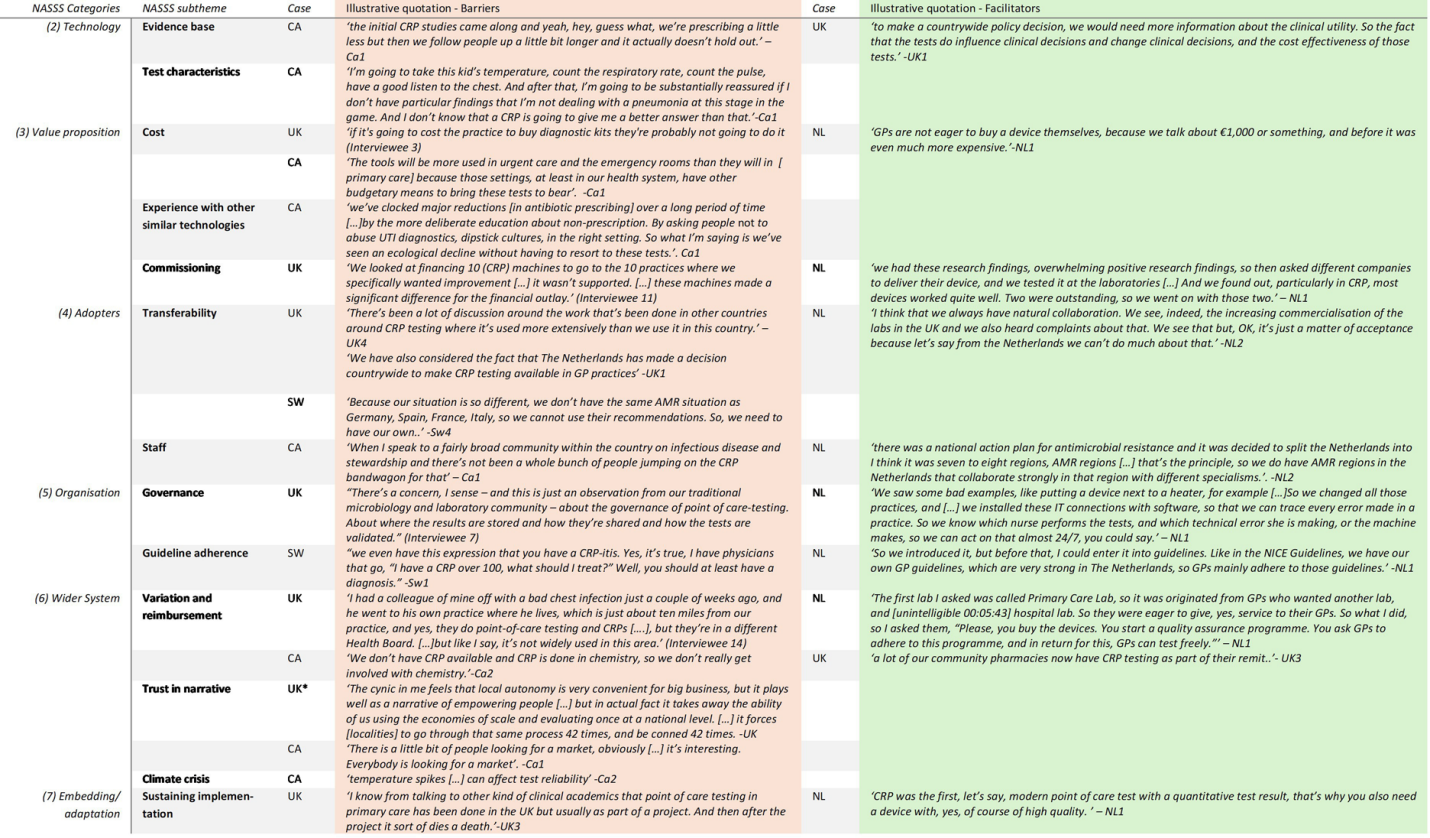
NASSS themes arising from the national-level interviews in Sweden, the Netherlands, the UK and Canada, by barriers and facilitators, supported by illustrative examples. *Interviewee requested additional protection so we have removed the link to even our anonymised identifiers, to decrease the risk that other quotes may provide a picture of who that interviewee might be. CA, Canada; NASSS, Non-adoption, Spread, Scale-up and Sustainability; NL, Netherlands; SW, Sweden.

The interviews were transcribed and manually coded with inductive and deductive codes. Further, where interviewee’s data were interpreted by coders to be an implementation barrier or facilitator, this was also coded. We grouped the coded NASSS themes into subthemes. The Topic Guide is included in the online supplemental appendix. Figure 1 provides more detail about the NASSS domain, the subthemes that arose and indicative data organised by barrier and facilitator, country and interviewee.

### Ethics approval

This study was reviewed and approved by the Health Research Authority (REC Ref: 22/HRA/3073) and the London School of Hygiene and Tropical Medicine Ethics Committee (LSHTM Ethics Ref: 27930). Ethics approvals covered online interviews on Zoom or Teams.

### Patient and public involvement

Patients and the public were involved in this evaluation when members of the public were asked about their experiences accessing primary care. Results from the patient and public involvement are published elsewhere.^22^

## Results

### Part 1: interviewees at the local and national level in the UK

In the UK, local and national interviewees reported barriers to implementing CRP testing across all six NASSS domains under investigation. Consistent positive views did not arise in a single domain, though the variation in local commissioning was by some mentioned as a facilitator of pilot and small-scale use of CRP testing. Others affirmed, however, that local commissioning was itself a strong barrier to CRP test implementation. Below, we delve into each NASSS domain in turn.

#### Technology (2)

Concerns about the evidence base and the performance characteristics of the CRP tests themselves arose as the dominant subtheme within this NASSS domain, among UK interviewees. One GP described the evidence base for CRP tests as ‘not robust enough’ (Interviewee 15), and another interviewee on a medicines optimisation team explained how CRP testing is only just being trialled in their area in urgent care centres and is not seen as sufficiently worthwhile to be made available in general practices (Interviewee 11). National policy-makers in general explained that the evidence base for CRP testing was not strong enough to warrant national-scale adoption:

[…] to make a countrywide policy decision, we would need more information about the clinical utility. So the fact that the tests do influence clinical decisions and change clinical decisions, and the cost effectiveness of those tests. (UK 1)

#### Value proposition (3)

Test cost, and which part of the organisation bears that cost, was reported by those working in GP practices and independent pharmacies as a barrier to diagnostic uptake. Two pharmacists identified cost and reimbursement mechanisms of the test as the main barrier to adoption (Interviewees 3 and 15).

A particular barrier to demonstrating the value of diagnostics in primary care was that there remains no central database listing which diagnostic tests are in use across the UK, and scarce formal information-sharing across integrated care boards (ICBs). This limits cross-locality learning and may exacerbate the assessment that CRP tests are poor value for money, since one laboratory contracting with a diagnostics company will have less leverage than were this conducted nationally, thereby impacting the cost and thence value proposition. One UK policymaker describes this barrier further:

[…] integrated care systems [ICSs] recommended that there was less central command and control, and more local autonomy and less national targets, etc. And so the direction of travel seems to be clear, even the establishment of integrated care systems made that clear, that we want—the Government wants local areas to have more control over dictating how money is spent, and what their priorities should be. But with that becomes a sort of a divide and conquer approach or mentality as far as the diagnostics companies are concerned, because they’ve got a smaller audience with less resources to investigate the value of the tests, and they have budgets and discretionary spend, and they may not be able to effectively evaluate the difference in value between different propositions of how they could spend that money. (UK 1)

The push for local autonomy in commissioning, in conjunction with a resource-constrained health system, has led to a scenario where even the most effective tests would struggle to be implemented. The governance systems in place for commissioning were also lacking, a theme that is discussed further in (5) and (6), below.

#### Adopters (4)

In classical Diffusion of Innovation theory, Rogers asserts that non-adopters look to early adopters, and adopt innovations where positive, transferable experiences have accrued.^20^ NASSS caveats the sinusoidal innovation uptake, and examines how and why non-adopters assess adopters as materially different, and insufficiently transferrable. National-level UK policy-makers cited other countries, including the Netherlands in their interviews, when assessing ‘transferability’.

There’s been a lot of discussion around the work that’s been done in other countries around CRP testing where it’s used more extensively than we use it in this country. (UK 4)

We have also considered the fact that The Netherlands has made a decision countrywide to make CRP testing available in GP practices. (UK 1)

Overall, however, CRP testing was seen to be ‘quite limited in its applicability in primary care’, according to a prescribing lead implementing national guidelines (Interviewee 13) and ‘not widespread [in our region]’ or ‘not something that we use or have used,’ according to an interviewee in primary care (Interviewee 9). Though some interviewees had heard of the tests being used elsewhere, or perhaps had experience of pilots, the overarching view was that the tests sat outside most interviewees’ experiences, preferences and field of view. The national level policy interviewees voiced their aim to improve the structural support available for CRP tests and similar point-of-care tests in primary care, though local level interviewees did not share the parallel aim of furthering test adoption.

#### Organisation (5) and the wider system (6)

Variation in reimbursement and commissioning mechanisms, as well as trust in diagnostic narratives and governance mechanisms, arose as particularly common and stark barriers identified by respondents in all case study sites, and also among national-level policymakers. One microbiologist explained how:

There’s a concern, I sense—and this is just an observation from our traditional microbiology and laboratory community—about the governance of point of care-testing. About where the results are stored and how they’re shared and how the tests are validated. (Interviewee 7)

The governance concerns voiced were not limited to particular teams given responsibility for test commissioning but were applied to the variation across the commissioning mechanisms in the system(s) as a whole. Pharmacies, GPs, urgent care centres and hospital laboratories conducting primary care tests (both on-site and off-site of the hospital) all commission and use (or do not use) tests according to their own preferences. However, there was scepticism regarding the governance of the commissioning process.

When probed about national/local governance, an interviewee in the UK who wanted to remain unattributed for their response to this particular question went one step further and said:

The cynic in me feels that local autonomy is very convenient for big business, but it plays well as a narrative of empowering people, and local democracy, but in actual fact it takes away the ability of us using the economies of scale and evaluating once at a national level.[…] it forces [localities] to go through that same process 42 times, and be conned 42 times.

The wording of this quotation is strong, and asserts that local commissioning (perhaps exacerbated by the lack of a centralised repository of data) did not simply hamper business case development on the adopter side, but was seen to favour diagnostics businesses at the expense of the publicly funded healthcare services in the UK.

#### Embedding and adaptation (7)

For the seventh element of the NASSS framework to be relevant, some pilots and trials need to occur in the first instance; indeed, there was some appetite for rolling out a pilot for CRP testing for specific conditions, such as urinary tract infections (UTIs) in general practice among our interviewees, but this had yet to be undertaken and no pilot funding had been identified, let alone follow-on funding (Interviewee 31). For those who had used CRP tests in primary care, they were either not subsequently embedded or only in early stages of adoption.

### Part 2: international comparisons

In order to inform the evaluation of the UK’s 2019–2024 AMR NAP’s diagnostic policy and to understand barriers and facilitators feeding into the diagnostic policies of the UK’s 2024–2029 AMR NAP, we sought to compare the CRP testing policies of Sweden, Canada and the Netherlands. There were mixed and positive responses in the two countries that had adopted CRP testing, whereas Canadian scepticism about CRP testing across all NASSS categories most reflected the UK context.

#### Technology (2)

Canadian respondents shared similar views about the evidence base for CRP testing to the UK respondents. One interviewee remarked that:

[…] the initial CRP studies came along and yeah, hey, guess what, we’re prescribing a little less but then we follow people up a little bit longer and it actually doesn’t hold out. (Ca 1)

In the Netherlands and Sweden, conversely, there was very little discussion about the evidence base on clinical or cost-effectiveness, and none at all about the evidence base being insufficient for action, perhaps unsurprisingly, since the policy decision to invest in CRP test implementation was taken decades ago. In Sweden, while the evidence base surrounding test characteristics was not discussed, the evidence base around postimplementation test uptake was a topic of strong discussion and is discussed in relation to embedding (7), below.

#### Value proposition (3)

Canadian interviewees viewed clinical judgement as superior to any of the CRP tests on the market, and all interviewees said CRP tests add relatively little value over clinical judgement. The medical experts within BC are very familiar with Northern European evaluations of CRP testing and are also aware of pilots and evaluations from Canada and the USA. One respondent explained:

[…] the question is, is [CRP] required to bring about those changes [to prescribing] or is it just another way to do it? If I sit down there and say, “Well wait a minute, I’m going to take this kid’s temperature, count the respiratory rate, count the pulse, have a good listen to the chest. And after that, I’m going to be substantially reassured, you know, if I don’t have particular findings that I’m not dealing with a pneumonia at this stage in the game.” And I don’t know that a CRP is going to give me a better answer than that. (Ca 1)

The Canadian case had the most reported ‘value proposition’ barriers and, critically, experiences with other similar technologies bled negatively into perceptions of CRP testing. The only place where Canadians reported seeing the value of CRP testing was in the secondary care context. The value proposition was hardly discussed by the Dutch and Swedish interviewees, apart from to explain why they organised their services as they did. One Dutch interviewee briefly explained that the system did not consider GPs having to purchase their own diagnostic equipment because ‘GPs are not eager to buy a device themselves, because we talk about €1000 or something, and before it was even much more expensive.’ (NL 1)

#### Adopters (4)

Discussions around the type of data and experiences that were used to inform views on adoption differed dramatically across the three sites. A Dutch interviewee explicitly described the importance for non-adopters to look to adopters and consider the transferability of the evidence that has been generated. They queried why the British did not consider the Dutch evidence to be sufficient:

…look at landscape of diagnostics SMEs I’m surprised with how easily new start-ups get given money in the UK when they could just be using the tests that we already have a decade of evidence on. (NL 2)

This Dutch interviewee believed that there was an implementation gap in the UK which lay at the end stage of implementation—when tests had strong evidence bases—and that, conversely, earlier stages in product development were favoured. They observed that the UK had a penchant for funding new diagnostics rather than implementing older ones. It cannot be assumed, however, that because the Netherlands adopted CRP testing, they were more likely to adopt yet other tests; the same Dutch respondent said that the GPs in the Netherlands like the system as it is, and are neither looking for the most recent technological advance, nor chasing small gains in diagnostic test accuracy.

Another Dutch interviewee was also critical about the Swedish CRP testing adoption, expressing multiple times that implementation of CRP testing in Sweden had ‘failed’ (NL 1) with patients expecting unnecessary CRP testing at most consultations for acute illness; this had been mitigated in the Netherlands, according to the interviewee, by a package of interventions, including the oversight and quality assurance of the tests being undertaken not by GPs but by laboratory staff, and also the training of GP staff in not just how to administer the tests, but when (NL 1).

In BC, there are few newer rapid diagnostics in use in primary care; stewardship interventions, not diagnostics, are attributed with achieving prescription reductions as high as 25%–50% over a 10-year period, for some individual antibiotics.^23^ Of the public and professional activities and resources made available to achieve these aims, no rapid diagnostics were commissioned as decision support aids.

When considering ‘transferable’ experiences with diagnostics, Canadian respondents were not only considering other countries’ CRP testing evidence bases, but also the transferability (or lack thereof) of learnings with other rapid diagnostics. For example, one respondent described:

we’ve clocked major reductions [in antibiotic prescribing] over a long period of time […] by the more deliberate education about non-prescription. By asking people not to abuse UTI diagnostics, dipstick cultures, in the right setting. So what I’m saying is we’ve seen an ecological decline without having to resort to these tests. (Ca 1)

Overall, then, transferable learnings in the three international contexts included other countries’ experiences, including negative experiences, but also countries’ own experiences with other rapid diagnostic tests, which in some cases fed into respondents’ views about CRP tests.

#### Organisation (5) and wider system (6)

Guideline adherence, a subtheme at the interface of organisation and systems, arose in all interviews with Swedish and Dutch experts.

There were divergent views among the Swedish interviewees on whether Swedish CRP test use in primary care was indeed guideline concordant. Within primary care teams, CRP tests are available and can be administered before a consultation with any professional, though the clinicians interviewed had divergent views about how well this works in practice. This interviewee reported limited (guideline concordant) use:

When we use something, we use CRP, but most of the time we don’t use—CRP is not in the algorithm, it’s just the findings of the patients […] I think doctors tend to take CRP a little more often with children, but otherwise they go by the clinical science (Sw 1)

However, the other Swedish AMR experts were more negative about guideline adherence, with examples of the over-use of CRP testing on patient entry to the healthcare centre, before triage or diagnosis and thus considered guideline discordant. The use of CRP tests in this manner in Sweden is seen as a policy failure, as one senior respondent described:

When it comes to CRP, we are a terrible CRP country. we take so much CRP and we’ve done it for ages. And there’s a lot of diseases where there’s no use for CRP, … you absolutely don’t need it for sinusitis, you don’t need it for tonsillitis. So, we’re stuck with the CRP and it’s not the CRP sampling that has reduced our antibiotic use. If we would have taken CRP on all our patients we would have used a lot more antibiotics, that’s what I believe. (Sw 2)

Conversely, Dutch interviewees stressed the importance of the wider system to the CRP test trajectory in the Netherlands, which they considered to be successful. Both interviewees described how primary care laboratories provide all testing resources and quality assurance for GP-based CRP testing, including primary care staff training; consumables reimbursement is provided by the healthcare insurers and the hardware is purchased for the practice by the primary care lab responsible for the test.^8^ In some cases, patients have to pay for their tests; in the case of the insurer reimbursement, this is provided directly to the laboratory responsible for the service, not to the GP. In this way, GPs can administer tests but do not hold responsibility for test maintenance, commissioning or reimbursement (20).

In spite of widespread positive descriptions of uptake across most NASSS domains among Dutch respondents, barriers were still reported to good practice. Typically, these related to quality assurance and IT systems. One interviewee explained:

…the IT components are a heavy burden because you have to combine those things if you want to introduce another test, another parameter. Sometimes you need other software to be connected, so at the moment, it’s for some labs, particularly the smaller ones, the burden to keep this system going, they feel like that. But it has also to do with the attitude, that they don’t really like this movement from central lab testing to a more decentralised system. (NL 2)

IT could be considered to be split among organisation (5) and wider system (6) as a subtheme, or even value proposition, depending on how IT systems are selected for and commissioned. What is more important than where IT systems sit within the NASSS classification is that it represents an interface issue; in the discussion, we describe how interface issues are poorly captured by the NASSS framework, and indeed frameworks in general.

Paying attention to subthemes from the inductive component of the coding, one interviewee in Canada described climate change as a wider systems issue that impacts diagnostic testing, and will do so even more in the future. This is because most rapid diagnostics are temperature-sensitive; one respondent described how the Heat Dome effect in Canada had invalidated many samples that had been tested using rapid technologies: ‘temperature spikes […] can affect test reliability’ (Ca 2). This is the only interview where climate change was directly cited as a barrier, though recent reports would indicate that this is likely to also be a concern for diagnostics globally in the near future, and would likely already be a cause for concern in facilities in the UK in the summer, as most do not have air conditioning.^24^ For example, most validation studies for CRP tests have been undertaken where the ambient or room temperature is 25° or lower.^25^

#### Embedding and adaptation (7)

Unlike the UK and Canada, where interviewees considered it too early or not relevant to consider embedding and adaptation, Dutch and Swedish respondents described how they were tracking postimplementation testing data, and in particular the proportion of tests that were being undertaken. It was this figure, primarily, that was being monitored for change. Respondents considered that increased testing was synonymous with a decrease in guideline adherence. In the Netherlands, guideline adherence was reported to be strong, with very little increase from the early phase of CRP implementation in the proportion of patients having CRP tests undertaken. In Sweden, however, that was not the case, with increased CRP testing in primary care over the past decade. One respondent explained that:

we even have this expression that you have a CRP-itis. Yes, it’s true, I have physicians that go, “I have a CRP over 100, what [condition] should I treat?” Well, you should at least have a diagnosis. (Sw 2)

In other words, CRP tests in Sweden are being more frequently used than they should, and in guideline discordant ways, including being used for conditions where they are likely to provide inaccurate diagnostic guidance, such as in ‘enteric pathogens’ (Sw 3).

## Discussion

This study aimed to support the evaluation of the UK’s 2019–2024 AMR NAP by exploring why uptake of CRP tests has not substantially progressed in primary care in the ten years since the UK AMR policies that first recommended their use.^5 17 26^ This diagnostic, so heavily favoured in the latter half of the 2010s, has failed to thrive in the UK’s policy and health service system. Decisions and activities of local-level and national clinical advisors and AMR policy-makers were constrained by a wide variety of contextual factors that influenced their views on the utility of CRP tests for AMR.

### Reflections within the UK

Though barriers to CRP test adoption were many and varied, and identified across all NASSS domains among local and national UK interviewees, all barriers are not created equally. While previous research has reflected on the importance of high quality tests, a strong evidence base and the early adopter-sustained adopter paradigm in this field,^14 26^ there are two unique findings arising out of this analysis within the UK context: a mistrust of businesses operating in the diagnostics space, and a parallel mistrust of extant public sector governance mechanisms. Taken together, these systems-level barriers create a critically poor environment for diagnostic implementation. Interviewees suggested key ways forward, including: tracking rapid diagnostic commissioning to determine whether investment is contributing to overall health improvement or creating (or exacerbating pre-existing) health disparities; and commissioning diagnostics nationally, in order to improve the value proposition while simultaneously exerting tighter control over the governance mechanisms in play. Not only do our findings align with the experimental data captured by the UK Office of National Statistics describing decreasing trust in public institutions year-on-year, but the interviewees’ proposed solutions align broadly with the OECD’s five public governance drivers to bolster trust in public institutions: integrity, responsiveness, reliability, openness and fairness.^27^ In particular, national or regional level diagnostics commissioning would improve the value proposition of tests while also improving reliability and fairness. Finally, regional-level procurement may have the added benefit over national-level procurement of tailoring the wider systems-level infrastructure (including QA and IT) to the region in question, according to rurality, presence of supportive infrastructure such as laboratory and diagnostics expertise, and available personnel.

### Reflections from Sweden, Canada and the Netherlands

Overall, the organisational environment and the wider systems carried enormous weight, both among countries where barriers to CRP test uptake proliferated, and where organisations and systems were considered to be facilitative to CRP test uptake.

It is striking that interviewees rarely equate test adoption with ‘successful’ implementation. In Canada and the UK, there were particularly strong concerns about clinical utility and CRP testing being aggressively marketed to policy-makers and clinicians. In Sweden, interviewees expressed regret that CRP testing was so widespread, and worry that CRP testing was fuelling increased, inappropriate prescribing, not reducing it. In the Netherlands, the system was deemed successful because the diagnostics are highly available but rarely used. Judicious testing is seen as success, not simply the availability of testing. Literature from other similar Scandinavian countries not under investigation casts doubt on the notion that CRP testing alone can significantly reduce antibiotic prescribing. One study in Norway found a non-significant reduction in antibiotic prescribing after CRP test uptake (in addition to increased cost), and a register-based study in Denmark found that GPs who used the most CRP tests prescribed more antibiotics than GPs who used the fewest CRP tests.^28 29^

Our interviewees within Sweden and the Netherlands both described Swedish CRP test implementation to be much less ‘successful’ than the Dutch case due to overtesting and resultant guideline-discordant overprescribing of antibiotics. These views align with published estimates of CRP tests being used in as many as 35%–65% of all Swedish primary care consultations for acute respiratory tract infection^7 8^ with the outcome of CRP testing not changing the clinical assessment of the physician and the antibiotic prescribing decision in 86% of the patients tested.^7^ Dutch CRP implementation has been more measured, with the proportion of GP consultations leading to a CRP test being an order of magnitude lower than in Sweden. One Dutch 3.5-year follow-up study after CRP implementation found that only 11 CRP tests were administered for 294 episodes of respiratory tract infection in the 203 patients in the CRP test group (3.7% of episodes).^30^ These findings align with a 10-year follow-up postimplementation analysis, which demonstrates that Dutch CRP testing has not risen over time.^31^ However, the interviewees do not critically reflect on the restrictive use of CRP in the Netherlands; it is conceivable that CRP testing in the Netherlands may be clinically insufficient; all we know is that it is more guideline concordant. Though we did not interrogate this ourselves in our study, we are aware of research into why Dutch GPs adhere strongly to guidelines.^32^

### Strengths and limitations

It could be argued that there was an imbalance between the UK data derived from 35 in-depth interviews and the smaller number of interviewees from the other countries involved in the study. This reflects the fact that the analysis was undertaken as part of an evaluation of the implementation of the UK AMR NAP and in order to put the UK situation into an international context. However, the non-UK interviewees were highly knowledgeable about the use of AMR diagnostics in their systems. In addition, we reviewed policy documents from each country to bolster the information from interviewees. A strength of this study was the use of the NASSS framework, which helped to structure the analysis. However, the study hints at the important levers of power dynamics between and among policymakers, clinicians and industry; NASSS may not be best placed to support further analysis in these fields. Moreover, for interface issues, such as IT interoperability, interprofessional negotiations or tensions, quality improvement or even transferring lessons among settings, NASSS may not provide the most support. While ‘transferability’ is preserved by both Diffusion of Innovation theory and NASSS as a characteristic of systems which adopt technologies, the interactive or interorganisational characteristics of operationalising transferability remain poorly formalised in this framework. Complex systems theory, which in fairness is encouraged by NASSS, could perhaps better capture these unpredictable or interactive wider systems effects.^21^

### Conclusions

The UNGA has encouraged diagnostic testing, including CRP tests, as a way of reducing the inappropriate use of antibiotics outside of the hospital setting. However, it is not self-evident that more testing is necessarily a goal to be uncritically pursued. This study shows that there are legitimate concerns about the value of such tests in day-to-day primary care as well as a range of implementation barriers that need to be overcome if testing is to be made widely available even in already well-organised healthcare systems. In addition, clinicians need to be encouraged and enabled to follow the prevailing guidelines on the appropriate use of CRP testing despite the fact that these currently rest on a far-from-perfect evidence base.

## Supplementary material

10.1136/bmjopen-2024-095059 online supplemental file 1

## Data Availability

Data are available on reasonable request.
