# Supplementary material for: C-reactive protein diagnostic test uptake in primary care: a qualitative study of the UK’s 2019–2024 AMR National Action Plan and lessons learnt from Sweden, the Netherlands and British Columbia
Source: BMJ Open. 2025 Aug 31;15(8):e095059. doi: 10.1136/bmjopen-2024-095059 (PMC12406930; doi:10.1136/bmjopen-2024-095059)
Supplement: online supplemental file 1 [file bmjopen-15-8-s001.docx]

Appendix 1: Topic Guide

**Draft Topic Guide – local actors**

**[Note – only relevant sections of the topic guide will be used for each interviewee]**

**Introductions and consent**

**About your involvement in AMR**

- Can you please describe your role in [organisation]
- Can you tell me about any initiatives to do with Anti-microbial use and resistance that you are involved in? (*if policy role)*
- Can you tell me about any aspects of your work that are relevant to anti-microbial use and resistance? (*if practice role*)

**Prescribing (human health)**

- Can you describe any local initiatives about prescribing antibiotics that have been put in place over the last few years?
- Examples might be feedback/audits for GPs; education (courses, etc); public / GP awareness raising, e.g. posters, radio, social media; any other stewardship initiatives; diagnostics/POC tests
- Has 'X' had an impact on prescribing? In what way has there been an impact? How do you know?
- How do you/your colleagues/patients feel about [the initiative]? Do you like/dislike it? Why/why not?
- Have there been any unintended positive benefits/negative consequences of [the initiative] that you didn’t predict?
- Can you describe any (other) changes in how you prescribe antibiotics over the last few years?

**Use of diagnostic tools in [setting]**

- Can you describe any diagnostic tools that are used in the hospital?
- Some common tests include the tests for MRSA, TB, or gram-negative bacterial infections.
- Other tests include bacterial vs. viral tests
- Other tests include covid-19 tests
- Can you describe how those tests are used in the hospital?
- Can you describe any difficulties with using those tests?
- Can you describe any changes to the clinical process that resulted due to these tests?
- Have you been involved in developing a business case for tests like these?
- What happened, decision, feedback?
- If there was a test for resistance available and you felt it represented value for money, do you think your organisation would purchase it?

**Use of diagnostic tools in primary care**

- Can you describe any diagnostics / point-of-care tests that are used with the aim of reducing antibiotic prescribing in primary care (or identifying specific pathogens), e.g. CRP tests?
- How long have you used [test]? Why did you start to use this test?
- Has 'X' had an impact on prescribing? In what way has there been an impact? How do you know?
- How do you/your colleagues/patients feel about [the test]? Do you/they like/dislike it? Why/why not?
- Are there any issues with using the test? E.g. difficult to use, expensive, etc
- Have there been any unintended positive benefits/negative consequences of [the test] that you didn’t predict?

**Wrap up**

- Can you think of specific local or regional reports or documents on prescribing or guidance documents that would be helpful for me to read?
- Is there anything else you would like to tell me about your work on AMR?
- Is there anyone that it would be especially important for us to talk to at this point? Any documents that would be relevant to our study?
