# Supplementary material for: C-reactive protein diagnostic test uptake in primary care: a qualitative study of the UK’s 2019–2024 AMR National Action Plan and lessons learnt from Sweden, the Netherlands and British Columbia
Source: BMJ Open. 2025 Aug 31;15(8):e095059. doi: 10.1136/bmjopen-2024-095059 (PMC12406930; doi:10.1136/bmjopen-2024-095059)
Supplement: Supplementary file 2 [file bmjopen-2024-095059.reviewer_comments.pdf]

PEER REVIEW HISTORY

BMJ Open publishes all reviews undertaken for accepted manuscripts. Reviewers are asked to complete a checklist review form and are provided with free text boxes to elaborate on their assessment. These free text comments are reproduced below.

ARTICLE DETAILS

Title (Provisional)

C-reactive protein diagnostic test uptake in primary care: a qualitative study of the UK's 2019-2024 AMR National Action Plan and lessons learned from Sweden, the Netherlands, and British Columbia

Authors

Glover, Rebecca E; Pacho, Agata; Mays, Nicholas

VERSION 1 - REVIEW

**Reviewer**	**1**
**Name**	**Borek , Aleksandra**
**Affiliation**	**SWPS University, Faculty of Psychology**
**Date**	**07-Jan-2025**
**COI**	**None**

This qualitative study explored factors that affected the uptake of CRP POCT in the UK and selected comparison countries. It tackles an important and interesting topic, using an appropriate qualitative approach and a relevant theoretical framework. However, the quality of the paper and the reporting could be improved; at present, I'm a bit concerned about the overall value of the paper and the quality of the study.

My main concerns relate to:

1. Comprehensive reporting – the methods of recruiting participants and collecting and analysing the data are not reported with sufficient transparency that would allow replication. I would recommend that the Authors check and comply with one of the reporting guidelines for qualitative research.

2. Depth and novelty of the findings – the results are reported on a somewhat superficial level, without sufficient nuances and details. The data seems to be summarised rather than fully synthesised. For example, I'm unsure whether the sub-headings are supposed to constitute themes or topics, and how they map with the domains from the NASSS framework. The barriers to using/implementing CRP POCTs that are reported are not novel and have been reported across many studies, including from the countries included in this

study. This paper could be more valuable if it drew out the comparisons between countries and synthesised them a bit more clearly into overarching themes.

3. How this study fits with the wider literature – the introduction and discussion are a bit thin. I would like to know a bit more about how exactly this study and paper fit with the wider evaluation of the NAP. Was this a separate sub-study or was the data collected as part of the evaluation re-analysed for this paper? The discussion would benefit from more comparison with the (fairly vast) literature on POCTs and their implementation to more clearly identify similarities and differences with the existing literature.

Below are my more detailed comments related to the issues mentioned above.

Abstract

- The abbreviation CRP would be better replaced with CRP POCT or CRP tests, e.g. in 'CRP adoption' or 'CRPs are unlikely to be implemented' (it's the POCTs that are adopted or implemented). The same comment applies to other uses of 'CRP' in the paper (e.g. p. 8, line 44).

- P. 5, like 35-6 – WHO European region countries – the countries listed should include the UK rather than Canada.

- P. 5 line 52 – what is meant or captured by AMR rates as influencing the selection of countries? Appendix 1 doesn't mention AMR rates but rather antibiotic use – should antibiotic use (rather than AMR) be mentioned here?

- Table 1 caption– should be the NASSS framework; please proofread the table to fix punctuation errors/discrepancies; it also looks like a different font was used across the table.

- P. 7, the phrase 'such as Rogers'' could be separated with two commas and it would be useful to include references both to the NASSS framework and Rogers' theory in the sentence.

- P. 7, line 44 – table 1 should be table 2.

Recruitment of participants:

- How were potential participants informed about the study (i.e., how was the study advertised, and how were potential participants reached)? Were they approached directly or through gatekeepers?

- Were all potential/interested participants included or were they purposefully sampled?

Data collection:

- Could the Authors provide a topic guide or interview schedule as an appendix?

- What languages were the interviews conducted in the Netherlands and Sweden?

Analysis:

- How exactly were the transcripts analysed? What does it mean that they were 'thematically coded'? The phrase implies thematic analysis but not quite because in Thematic Analysis data is first coded and themes are subsequently developed.

- How exactly and in what order were the data coded both inductively and deductively? I.e. were the data first coded inductively and then the NASSS concepts applied to the inductively-developed categories? Or was this done in some other way?

- Was any software used to code the transcripts and manage the data or were transcripts manually coded/annotated 'on paper'?

- Were the data from different countries analysed together? How were comparisons between countries made?

- How long were the interviews?

- Who collected and analysed the data? Was it all done by one person or was a team involved, and in what ways? It would be helpful to also understand a bit about the background of the researchers (e.g. a short section on positionality/reflexivity about what the researchers brought to the project).

Results:

- It would help if more information was provided about the interviewees rather than only attributed to numbered interviewees. E.g., p. 8, line 51 – what role did interviewee 31 have? I'd recommend either listing all interviewees with their numbers, region and role in a table or providing this information in brackets each time an interviewee is cited or referred to.

- The cost of POCTs and reimbursement mechanisms are mentioned under 'evidence gaps' as well as reported below under the next sub-heading. Could it perhaps be clarified why it is reported in both places or moved to just one section?

- 'Climate crisis' – why is this being introduced specifically as a barrier to implementing' RDTs whereas no sub-heading is introduced to capture other types of factors?

- Where policy is mentioned in the Results, where does this data come from and how was it analysed? Does it all come from interviews alone? If not (e.g. if policy-related findings reported in the Results come directly from literature, then this should be described in the Methods).

Overall:

- I'd suggest ensuring more consistent use of abbreviations (e.g. POCTs, RTDs) after they are introduced, introducing all abbreviations at first use (e.g. BC, QA), and more careful proofreading (especially regarding punctuation).

- There's an inconsistency in how interviewees are referred to (e.g. on p. 8 and UK1 on p. 9).

- P. 12, line 6 – a word is missing (more frequently used?).

- Subheading types are used inconsistently and at times it’s unclear which level of a heading is used.

**Reviewer**	**2**
**Name**	**Verbakel, Jan**
**Affiliation**	**University of Oxford, Nuffield Department of Primary Care Health Sciences**
**Date**	**21-Feb-2025**
**COI**	**None**

Dear Editor,

I would like to thank you for the opportunity to review the paper by Glover et al., entitled: "C-reactive protein diagnostic uptake in primary care: a qualitative study of the UK’s 2019-2024 AMR National Action Plan and lessons learned from Sweden, the Netherlands, and British Columbia".

This manuscript presents a qualitative analysis of the implementation of C-reactive protein (CRP) testing in primary care across four countries, with a focus on antimicrobial resistance (AMR) policies. The study is well-structured, methodologically sound, and provides valuable insights into how policy, financing, and professional attitudes shape diagnostic adoption.

However, there are several areas that require further clarification and refinement. In particular, the manuscript would benefit from more elaboration on key assumptions, a more critical discussion of the Dutch model, and a stronger synthesis of existing evidence on CRP effectiveness.

I have listed my questions below:

- The use of the NASSS framework is well justified, but its limitations in this context are not discussed.

- NASSS is effective in mapping complexity in health technology adoption, but does it fully capture the financial and political drivers behind CRP implementation?

- A discussion of health technology assessment (HTA) principles could strengthen the analysis, particularly in evaluating cost-effectiveness and decision-making processes around CRP uptake.

- The study hints at power dynamics between policymakers, clinicians, and industry—how well does NASSS account for this?

- The Dutch model is portrayed as the most successful, but the discussion lacks critical reflection on its potential downsides.

Could the restrictive use of CRP in the Netherlands lead to underuse? Are there patient groups that may benefit from wider availability of CRP testing?

The Dutch system is structured around insurer-funded primary care labs—how transferable is this model to the UK and Canada, which have different financing structures?

While Dutch guideline adherence is strong, how much of this is cultural vs. policy-driven? Are there mechanisms that actively enforce appropriate use, or is it self-regulated?

- Do Dutch and Swedish clinicians interact with CRP manufacturers differently, or are their purchasing behaviors simply shaped by different financing models?

Have commercial actors influenced CRP test pricing, quality standards, or training programs in these countries?

Would centralizing diagnostic commissioning in the UK improve uptake and guideline adherence, or could it introduce new bureaucratic hurdles?

Could regional procurement models provide an easier alternative?

- Are Canadian clinicians truly evidence-driven, or is resistance to CRP testing shaped by other things?

- Finally, is there any data on the long term impact of point-of-care CRP on AMR? Are there examples from other countries (e.g., Denmark, Norway) where CRP adoption has been linked to reductions in AMR rates?

VERSION 1 - AUTHOR RESPONSE

2. Reviewer 1

-Comprehensive reporting – the methods of recruiting participants and collecting and analysing the data are not reported with sufficient transparency that would allow replication. I would recommend that the Authors check and comply with one of the reporting guidelines for qualitative research.

Thank you, we have complied with the SRQR checklist, and have uploaded this with the revision.

-Depth and novelty of the findings – the results are reported on a somewhat superficial level, without sufficient nuances and details. The data seems to be summarised rather than fully synthesised. For example, I'm unsure whether the sub-headings are supposed to constitute themes or topics, and how they map with the domains from the NASSS framework. The barriers to using/implementing CRP POCTs that are reported are not novel and have been reported across many studies, including from the countries included in this study. This paper could be more valuable if it drew out the comparisons between countries and synthesised them a bit more clearly into overarching themes.

Thank you for this comment, which we agree with. We have modified the paper substantially in line with your constructive critique. The first part of the results section is now entirely reported by NASSS domain (grouping local and national UK interviewees), and the second part of the results also reports findings across NASSS domains, rather than by country. Instead, the country-specific findings are brought into conversation with one another within the NASSS domains. Thank you for this challenge, which we believe has strengthened the paper. (We have also added detail on how deductive themes and inductive subthemes arose from the data. Finally, we describe how we coded barriers and facilitators as an additional analysis layer.

How this study fits with the wider literature – the introduction and discussion are a bit thin. I would like to know a bit more about how exactly this study and paper fit with the wider evaluation of the NAP. Was this a separate sub-study or was the data collected as part of the evaluation re-analysed for this paper? The discussion would benefit from more comparison with the (fairly vast) literature on POCTs and their implementation to more clearly identify similarities and differences with the existing literature.

Thank you for this important point. We have included substantially more literature in the discussion section, including about the uptake of POCTs in comparable primary care settings. We have also clarified in the methods that the qualitative interview data was collected across a number of themes for the wider evaluation, but the data on CRP testing was analysed for this paper specifically.

Below are my more detailed comments related to the issues mentioned above.

Abstract
- The abbreviation CRP would be better replaced with CRP POCT or CRP tests, e.g. in 'CRP adoption' or 'CRPs are unlikely to be implemented' (it's the POCTs that are adopted or implemented). The same comment applies to other uses of 'CRP' in the paper (e.g. p. 8, line 44).

Done – I have used 'CRP test' or 'CRP testing' throughout now

- P. 5, like 35-6 – WHO European region countries – the countries listed should include the UK rather than Canada.

Thank you for catching this – have amended

- P. 5 line 52 – what is meant or captured by AMR rates as influencing the selection of countries? Appendix 1 doesn't mention AMR rates but rather antibiotic use – should antibiotic use (rather than AMR) be mentioned here?

Thank you, this has been amended.

- Table 1 caption– should be the NASSS framework; please proofread the table to fix punctuation errors/discrepancies; it also looks like a different font was used across the table.

Great catch! We have now amended.

- P. 7, the phrase 'such as Rogers'' could be separated with two commas and it would be useful to include references both to the NASSS framework and Rogers' theory in the sentence.
- P. 7, line 44 – table 1 should be table 2.

Thank you, I believe we have actioned most of these minor comments now.

Recruitment of participants:
- How were potential participants informed about the study (i.e., how was the study advertised, and how were potential participants reached)? Were they approached directly or through gatekeepers?

We directly approached all our participants via email and conducted our own recruitment.

- Were all potential/interested participants included or were they purposefully sampled?

The case study sites, included country comparators, and individual interviewees were all purposively sampled, as described in the text (pp 5/6).

Data collection:
- Could the Authors provide a topic guide or interview schedule as an appendix?

Added to Appendix 2.

- What languages were the interviews conducted in the Netherlands and Sweden?

English (have added this detail into 'interview data collection', p7).

Analysis:
- How exactly were the transcripts analysed? What does it mean that they were 'thematically coded'? The phrase implies thematic analysis but not quite because in Thematic Analysis data is first coded and themes are subsequently developed.

Thank you for catching this typo. We have amended this now.

- How exactly and in what order were the data coded both inductively and deductively? I.e. were the data first coded inductively and then the NASSS concepts applied to the inductively-developed categories? Or was this done in some other way?

We used the NASSS framework to create the topic guide (as described in the 'development of the topic guide' section) and have added more detail on coding throughout, with thanks. In brief, inductive codes arose within each NASSS framework, leading to the development of the subthemes in Figure 1 and reported throughout the results section.

- Was any software used to code the transcripts and manage the data or were transcripts manually coded/annotated 'on paper'?

As coming from a critical sociological background, researchers on the study consider NVivo and other coding softwares to create distances from the data, and so do not typically consider this best practice. We code each transcript on the document we get back from the transcribers after close readings and rereadings of the text.

- Were the data from different countries analysed together? How were comparisons between countries made?

Due to your comments above, we have completely rewritten the comparative country analysis and believe there is much more information about the comparisons in the results section (especially part 2), thank you.

- How long were the interviews?

Great catch, we have now added the times into the 'interview data collection' section.

- Who collected and analysed the data? Was it all done by one person or was a team involved, and in what ways? It would be helpful to also understand a bit about the background of the researchers (e.g. a short section on positionality/reflexivity about what the researchers brought to the project).

Thank you we have added a sentence about the coding, recoding, and analysis, and included a positionality/reflexivity statement.

Results:

- It would help if more information was provided about the interviewees rather than only attributed to numbered interviewees. E.g., p. 8, line 51 – what role did interviewee 31 have? I'd recommend either listing all interviewees with their numbers, region and role in a table or providing this information in brackets each time an interviewee is cited or referred to.

  Unfortunately, many of these interviewees are high-level policymakers. We consider the risk of pseudonymity high in this research, but have clarified why this is particularly the case in the article, and referenced the anonymity methods we have chosen to follow in our article.

- The cost of POCTs and reimbursement mechanisms are mentioned under 'evidence gaps' as well as reported below under the next sub-heading. Could it perhaps be clarified why it is reported in both places or moved to just one section?

  Due to word limit constraints, I previously collapsed NASSS domains together, but agree that this was not clear. I have now extended the word count in order to respond to your comment, and it should now be clear the links between NASSS domains, and subthemes, since the same domains in Figure 1 (and Table 1) are used to structure both Part 1 and Part 2 of the results. Thank you for pushing us to restructure our work – it has helped!

- 'Climate crisis' – why is this being introduced specifically as a barrier to implementing' RDTs whereas no sub-heading is introduced to capture other types of factors?

  Now that the results section is restructured, climate crisis is a paragraph within part 2, under 'wider system'. I felt it was important to pay attention to divergent themes, and while this only arose in one interview, it is likely to be more pertinent over time, so wanted to highlight in the appropriate results section. I hope this is clearer now.

- Where policy is mentioned in the Results, where does this data come from and how was it analysed? Does it all come from interviews alone? If not (e.g. if policy-related findings reported in the Results come directly from literature, then this should be described in the Methods).

  Thank you for this opportunity – we have combined the policy in the introduction section, so that should be clearer now.

Overall:

- I'd suggest ensuring more consistent use of abbreviations (e.g. POCTs, RTDs) after they are introduced, introducing all abbreviations at first use (e.g. BC, QA), and more careful proofreading (especially regarding punctuation).

Thank you – this has been standardised.

- There's an inconsistency in how interviewees are referred to (e.g. on p. 8 and UK1 on p. 9). *Changed, with thanks*
- P. 12, line 6 – a word is missing (more frequently used?) *Added*
- Subheading types are used inconsistently and at times it's unclear which level of a heading is used. *I think I have fixed this.*

Reviewer: 2
Dr. Jan Verbakel, University of Oxford, KU Leuven
Comments to the Author:
Dear Editor,
I would like to thank you for the opportunity to review the paper by Glover et al., entitled: "C-reactive protein diagnostic uptake in primary care: a qualitative study of the UK's 2019-2024 AMR National Action Plan and lessons learned from Sweden, the Netherlands, and British Columbia".
This manuscript presents a qualitative analysis of the implementation of C-reactive protein (CRP) testing in primary care across four countries, with a focus on antimicrobial resistance (AMR) policies. The study is well-structured, methodologically sound, and provides valuable insights into how policy, financing, and professional attitudes shape diagnostic adoption.

Thank you!

However, there are several areas that require further clarification and refinement. In particular, the manuscript would benefit from more elaboration on key assumptions, a more critical discussion of the Dutch model, and a stronger synthesis of existing evidence on CRP effectiveness.

I have listed my questions below:
- The use of the NASSS framework is well justified, but its limitations in this context are not discussed.
- NASSS is effective in mapping complexity in health technology adoption, but does it fully capture the financial and political drivers behind CRP implementation?

Good question. NASSS makes provision for financial and political drivers in its 'wider system' (section 6) but you're right that in the framework it does not explicitly mention financial/political drivers (though the paper does). We have added some text into the methods section describing NASSS, and a paragraph in the discussion about the limitations of NASSS, in particular around (a) interface issues and power relationships.

- A discussion of health technology assessment (HTA) principles could strengthen the analysis, particularly in evaluating cost-effectiveness and decision-making processes around CRP uptake.

I agree that this is very important, but we have tried to keep our study focused on what our respondents say is important to their decision making (eg the literature more broadly), rather than what systems exist to make CE/HTA decisions. However, we have added a detailed paragraph on the policies governing CRP testing in each of the four countries in the introduction to better explain the context, which we agree was insufficient.

- The study hints at power dynamics between policymakers, clinicians, and industry—how well does NASSS account for this?

You're right, this is a limitation of NASSS. Well-considered point – we have added this to our limitations. We acknowledge this limitation, and recommend future research using more complex systems theory might mitigate this risk in future.

- The Dutch model is portrayed as the most successful, but the discussion lacks critical reflection on its potential downsides.
Could the restrictive use of CRP in the Netherlands lead to underuse? Are there patient groups that may benefit from wider availability of CRP testing?

I think you're right, actually, this was a blind spot for the research team, generally – we have added this point to our discussion.

The Dutch system is structured around insurer-funded primary care labs—how transferable is this model to the UK and Canada, which have different financing structures?

Not very – we have added this to the discussion.

While Dutch guideline adherence is strong, how much of this is cultural vs. policy-driven? Are there mechanisms that actively enforce appropriate use, or is it self-regulated?

We agree – thank you for pointing out our weakness and underlying assumptions here! I have taken on board this comment in particular. I have included instances throughout this paper critically appraising 'successful implementation', and disentangling a little bit better 'increased test use' vs 'guideline concordant/discordant behaviour'. I really appreciate you clarifying my assumptions – and you are right, there might be a risk of undertesting in the Dutch system. I don't know the cultural literature in this field, but have added a reference to literature about Dutch guideline adherence in CRP testing.

- Do Dutch and Swedish clinicians interact with CRP manufacturers differently, or are their purchasing behaviours simply shaped by different financing models?
Have commercial actors influenced CRP test pricing, quality standards, or training programs in these countries?

I am not sure I would want to speculate, since this did not arise in the data, but I have pushed this more in the UK section – as you will see I have added more quotations about the UK and Canadian policymakers' scepticism about governance in commissioning in this way.

Would centralizing diagnostic commissioning in the UK improve uptake and guideline adherence, or could it introduce new bureaucratic hurdles?

Probably both!

Could regional procurement models provide an easier alternative?

Yes, possibly, although I haven't previously given a regional approach much though. My understanding is that regional- (and local-)level commissioning is meant to be best suited for service commissioning where there could be credibly said to be population differences driving different healthcare needs, and the model of CRP testing that appears to be most effective is test availability within all primary care centres, but with rare usage only when the test will most likely inform clinician behaviour (eg in cases of uncertainty). Then again, you're right, regional commissioning could account for the different wider systems-level constellation of supportive infrastructure (QA and IT issues, allowing more rural/urban areas to commission differently, staffing). Alright, I'm convinced! I have added a line in the discussion to that effect. Thank you.

- Are Canadian clinicians truly evidence-driven, or is resistance to CRP testing shaped by other things?

I don't want to question their view of their evidence-based perspective – I am only commenting on what they reported to us. However, I have clarified this a bit in the text.

- Finally, is there any data on the long term impact of point-of-care CRP on AMR? Are there examples from other countries (e.g., Denmark, Norway) where CRP adoption has been linked to reductions in AMR rates?

I have added additional references in the discussion on this point for both Denmark and Norway. Thank you for such constructive comments throughout your peer review. I appreciated your thoughtful and incisive review, and hope that I have suitably addressed your comments and concerns throughout. I have particularly enjoyed thinking about the fair questions you have posed.

VERSION 2 - REVIEW

**Reviewer**	**1**
**Name**	**Borek , Aleksandra**
**Affiliation**	**SWPS University, Faculty of Psychology**
**Date**	**23-Apr-2025**
**COI**	

Thank you for addressing the comments and revising the manuscript. I especially appreciate you substantially rewriting the results section, which is now much better in my view – the NASSS domains and the cross-country comparisons (which are the strengths of this study) are now much clearer.

However, I still have a few minor comments on the revised manuscript.

- Title – I'd like to suggest perhaps adding 'test' in the title as 'diagnostic tests' is a common phrase and might help readers find the paper: 'C-reactive protein diagnostic test uptake in primary care'.

- Please spell out and add a reference where the NASSS framework is first referred to (p. 5); also in the same sentence, perhaps rephrase to 'to better guide CRP test implementation'

- P. 6, please spell out ICB on first use.

- P. 6 – please include the references to the NASSS framework and the Diffusion of Innovation theory in the text.

- Data analysis section: In the revised version, you still report for 'themes to be coded'. I don't want to be nit-picking but, as a qualitative researcher myself, I would like to encourage more precise reporting of qualitative analysis methods. Themes are not codes; themes are the result of the analysis. So, in my view, the first sentence would be more accurately reported as 'and transcripts were manually coded using inductive and deductive codes'. Also, the sentence: 'We grouped the coded NASSS themes into subthemes, which arose inductively from the data' – please consider rephrasing as it doesn't make sense to me that you group themes (higher-level) into sub-themes (lower level). Do you mean that you re-coded the data initially coded using the NASSS-framework domains into more detailed inductive codes? Finally, themes do not 'arise' (nor do they 'emerge') – they are identified by the researchers. So please remove this misleading phrase.

- Figure 1 is not provided. I noticed also that in the SRQR you referred to 'Figure 2' – should this also be Figure 1? Or is there another figure that is not provided nor referred to in the paper?

- P. 9, ICB is defined here but has been used earlier so please define it on the first use and then use the abbreviation.

- P. 9, 'were found to be wanting' – unclear phrase, do you mean that they were wanted? Or maybe better: recommended?

- P. 11, 'lower or similar rates of AMR in humans' – for consistency and correctness, please change to 'antibiotic usage rates' (as was done earlier in the paper).

- P. 14, 'took pains to stress' – please consider rephrasing this informal wording. Perhaps 'stressed' alone is sufficient.

- Conclusions – please spell out 'UNGA'.

VERSION 2 - AUTHOR RESPONSE

Reviewer: 1
Dr. Aleksandra Borek , SWPS University
Comments to the Author:
Thank you for addressing the comments and revising the manuscript. I especially appreciate you substantially rewriting the results section, which is now much better in my view – the NASSS domains and the cross-country comparisons (which are the strengths of this study) are now much clearer.

Thank you!

However, I still have a few minor comments on the revised manuscript.

- Title – I'd like to suggest perhaps adding 'test' in the title as 'diagnostic tests' is a common phrase and might help readers find the paper: 'C-reactive protein diagnostic test uptake in primary care'.

Good idea, we have actioned this now.

- Please spell out and add a reference where the NASSS framework is first referred to (p. 5); also in the same sentence, perhaps rephrase to 'to better guide CRP test implementation'

This is actioned now.

- P. 6, please spell out ICB on first use
We have reflected that ICB is in fact not appropriate here since some sites in Scotland/Wales are not in fact ICBs. To avoid confusion, we have listed these earlier in the methods and added 'case study site' where it used to say ICB.
- P. 6 – please include the references to the NASSS framework and the Diffusion of Innovation theory in the text.

Done.

- Data analysis section: In the revised version, you still report for 'themes to be coded'. I don't want to be nit-picking but, as a qualitative researcher myself, I would like to encourage more precise reporting of qualitative analysis methods. Themes are not codes; themes are the result of the analysis. So, in my view, the first sentence would be more accurately reported as 'and transcripts were manually coded using inductive and deductive codes'. Also, the sentence: 'We grouped the coded NASSS themes into subthemes, which arose inductively from the data' – please consider rephrasing as it doesn't make sense to me that you group themes (higher-level) into sub-themes (lower level). Do you mean that you re-coded the data initially coded using the NASSS-framework domains into more detailed inductive codes? Finally, themes do not 'arise' (nor do they 'emerge') – they are identified by the researchers. So please remove this misleading phrase.

This phrase is now removed, and the 'themes coded' has been edited to simply say 'coded'.

- Figure 1 is not provided. I noticed also that in the SRQR you referred to 'Figure 2' – should this also be Figure 1? Or is there another figure that is not provided nor referred to in the paper?

There is Table 1 and Table 2, and Figure 1, you are right. I meant Figure 1. This is now amended.

- P. 9, ICB is defined here but has been used earlier so please define it on the first use and then use the abbreviation.

Because we removed ICB earlier we have left this here.

- P. 9, 'were found to be wanting' – unclear phrase, do you mean that they were wanted? Or maybe better: recommended?

'were found to be wanting' is standard English, however we have modified to 'lacking'.

- P. 11, 'lower or similar rates of AMR in humans' – for consistency and correctness, please change to 'antibiotic usage rates' (as was done earlier in the paper).

Fixed, thank you.

- P. 14, 'took pains to stress' – please consider rephrasing this informal wording. Perhaps 'stressed' alone is sufficient.

Fixed, thank you!

- Conclusions – please spell out ‘UNGA’.

UNGA was introduced in full and as an acronym in the introduction so we have kept it as such here.
